# Design and analysis of welding inspection robot

**DOI:** 10.1038/s41598-022-27209-4

**Published:** 2022-12-31

**Authors:** Pengyu Zhang, Ji Wang, Feng Zhang, Peiquan Xu, Leijun Li, Baoming Li

**Affiliations:** 1 School of Materials Science and Engineering, Shanghai University of Engineering Science, Shanghai, 201620 China; 2 Department of Chemical and Materials Engineering, University of Alberta, Edmonton, T6G 1H9 Canada; 3 Yanfeng Visteon Electronic Technology (Shanghai) Co., Ltd, Shanghai, 200235 China

**Keywords:** Mechanical engineering, Electrical and electronic engineering

## Abstract

Periodic inspection, commonly performed by a technician, of weld seam quality is important for assessing equipment reliability. To save labor costs and improve efficiency, an autonomous navigation and inspection robot is developed. The development process involves the design of chassis damping, target detection mechanism, control system, and algorithms. For performing weld inspection in complex, outdoor, environments, an algorithm is developed for the robot to avoid any obstacles. This algorithm for planning the inspection route is based on an improved timed-elastic-band (TEB) algorithm. The developed robot is capable of conducting inspection tasks in complex and dangerous environments efficiently and autonomously.

## Introduction

With the rapid developments, robots can perform simple or complex tasks in dangerous environments that are beyond people's reach. Today, the use of robots as work aids has become increasingly common in both industrial and consumer spaces. Robots can reduce labor costs, save time, improve safety, and improve the quality of work^1^. Robots also play a significant role in welding, such as spot welding for automotives, arc welding for bridge girders, and welding of polymer-matrix composites. Welding is widely used for metallic materials, from vessels and pipelines to bridges and railways^2^. Good weld quality can ensure the strength and toughness of the joints. During service, welds often will deteriorate, by corrosion and fatigue cracking, and result in structural failures. The most serious failures often involve welds in critical locations, where damage caused by corrosion leads to cracking, leakage, or bursting of vessels. In large-scale infrastructures where the welds deteriorate over a large area, it is impractical and cumbersome to inspect the welds using human labor. The use of robots becomes necessary and feasible for these cases^3^. The design of weld inspection robots revolves around two questions: how can the robot accurately reach the place where the weld is located, and how can the robot accurately inspect the weld.

Different solutions have been proposed by researchers to solve these essential weld-inspection robotic questions. A robotic climber with multiple breathing chambers for inspection was designed for the inspection of concrete walls^4^. The propulsion system consisted of three omnidirectional drive wheels with a great maneuverability. Combined with a vacuum system comprising seven controllable vacuum chambers and a large fluid reservoir operating system, the robot had pressure sensors and valves integrated for controls. Shang et al. introduced a method that utilized neodymium permanent magnets for bonding, giving the robot a payload-carrying capacity^5^. The arrangement of the magnets improved the ground clearance, enabling the robot to overcome obstacles. To be able to work on curved surfaces, a wheeled robot with two articulated segments was designed, which had the advantages of high speed and good maneuverability.

A robot with magnetic wheels and vision sensors for defect detection was designed, and the drive mechanisms of inspection robots had included three forms: a track, a wheel, and a leg^6,7^. Adsorption or magnet suction affixed to the location of detection had been the most common for welding inspecting robots. The sensors would detect the weld seam and then extracts the weld seam geometry, and correct the robot's position in real time^8,9^, but the degree of automation was low and the requirements for sensor accuracy were high.

Inspection robots have recently evolved to become autonomous and semi-autonomous, for saving inspection time and reducing labor costs. Nita and other researchers^10^ have studied a semi-autonomous tracked inspection robot to detect defects in building ceilings. The developed inspection robots were equipped with wireless cameras and data processing functions, which could provide valuable information for the repair of the damaged structures. The inspection robot was able to assess the damage without the need for an engineer on site. Krenich^11^ designed a six-axis robot that could move autonomously for inspection, while a human could also inspect around the weld seam with a camera carried by the robot. Bruzzone^12^ introduced a mobile robot with a hybrid wheel and leg design, which featured wheels that could roll on flat grounds at a high speed, while the legs enabled the robot to avoid obstacles and to climb hills^13^.

Detection algorithms were developed for corrosion and cracking in aged welds. The use of advanced vision algorithms based on deep learning and machine learning made it possible to detect and recognize the defects. An algorithm based on an improved Gaussian mixture model for weld seam detection and classification was introduced by Sun et al.^14^ It could classify the identify the weld defects with a high accuracy and in real-time. Li et al. proposed a deep learning-based algorithm for weld seam image recognition^15^, which accelerated the neural training on several thousand images of the welds. The disadvantage of this algorithm was that it was too computationally intensive and hardware demanding. Yang et al. proposed a method to improve the defect localization of U-net mesh to improve the automatic localization accuracy for weld detection^16^. For low-cost robot development using low- to mid-cost main control boards, less computationally intensive algorithms need to be developed to achieve the identification and localization of weld defects.

Based on the above survey of literature, the objectives of the present research include: (1) Design of an autonomous weld inspection robot and its control system. The robot can achieve autonomous weld inspection, and can pass smoothly between narrow spaces with less time while autonomously avoiding obstacles. (2) Design of a yolo5-based target algorithm for weld seam detection and identification in complex environments.

**Figure 1:**
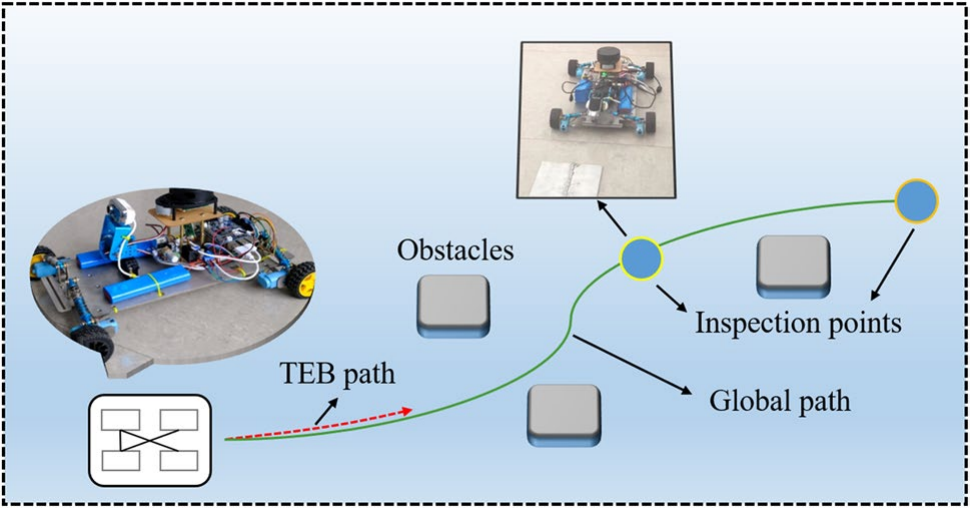
Work overview of weld inspection robot.

Figure 1 shows an overview of the scope-of-work for the autonomous weld inspection robot, which dodges obstacles in a complex environment and performs inspection of the weld seam at the inspection point.

## Design layout

The design of the autonomous inspection robot is divided into four different components: mechanical structure design, chassis motion control design, vision detection system design, and control system architecture design.

### Structural design of the robot

**Figure 2:**
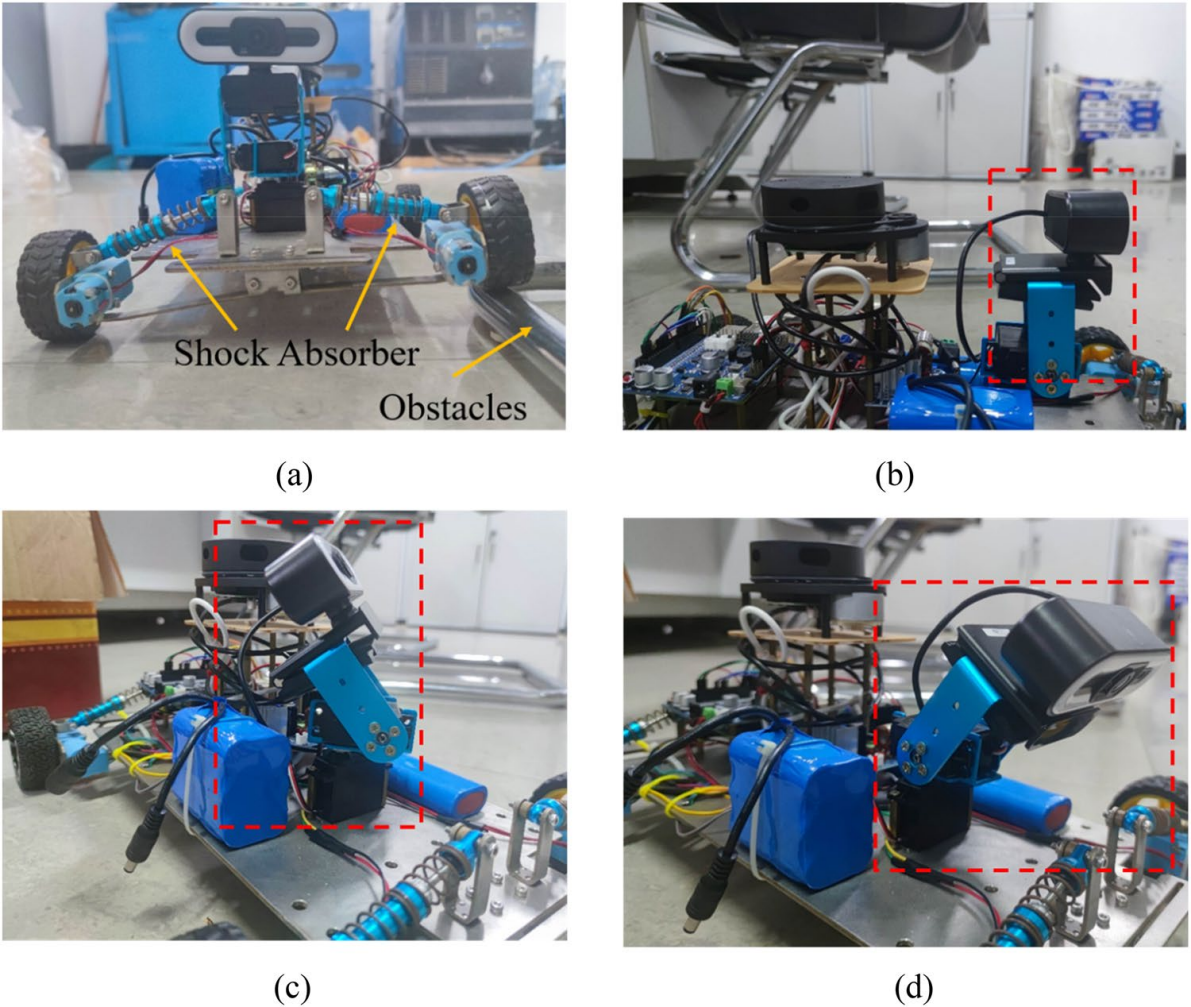
Status of the shock absorber and visual head.

**Figure 3:**
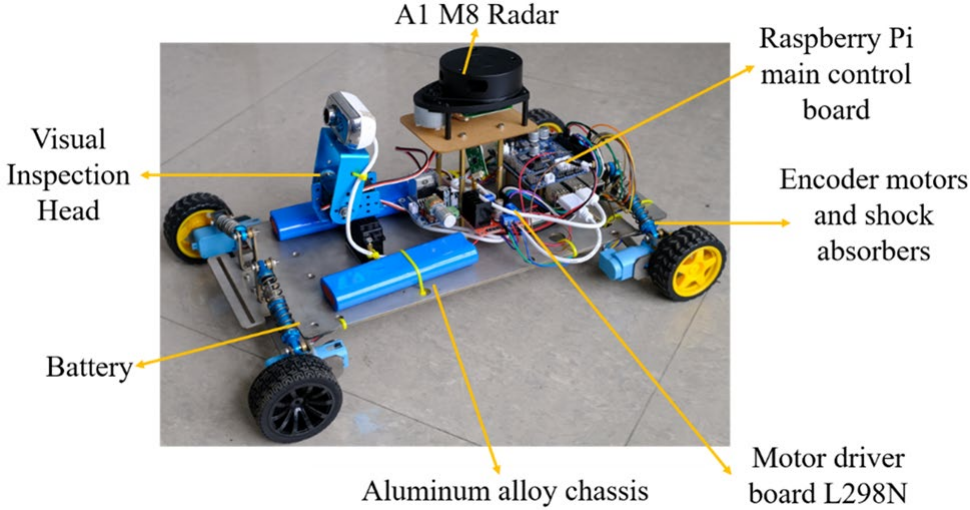
Structural design of the robot.

An aluminum chassis is used as the main frame of the robot, and each motor is individually attached to the chassis through a motor mounting plate. Shock absorbers are placed between the motor mounting plate and the chassis to reduce the vibration of the robot and to help it pass through obstacles as shown in Fig. 2a, so that the robot maintains its posture when the wheels cross over the obstacles. Each wheel is controlled by a separate gear motor for proper power distribution as well as flexible control. Encoders and a 1:90 gear ratio allow large torques to be transmitted to the wheels. High precision motors provide precise feedback of speed and position information. The four-wheel drive allows for fast turning and easier passage through complex roads. Due to the high mounting position of the radar, smaller obstacles cannot be detected, so ultrasonic sensors and infrared sensors are used to solve the collision problems caused by the blind spots of the radar. The main control system is mounted on the chassis and equipped with a sensor-radar with obstacle detection functions. For weld seam detection, the robot's detection system consists of a camera, two servos, two brackets and several bolts. Adjusting the orientation of the servos allows for multi-directional detection as shown in Fig. 2b–d. The information received by the inspection system is transmitted to a PC, on which the inspection information can be viewed and analyzed remotely. The complete structural design of the robot is shown in Fig. 3.

### Chassis motion control design

Popular control models for chassis drive include the Wheel Differential model, Ackerman model, and Omnidirectional model. The turning radius of the Ackerman model cannot be 0, which does not allow the robot to turn in narrow space. The Omnidirectional model needs to use McNamee wheels instead of the common wheels. In addition, the gaps between each small wheel of the McNamee wheel are liable to be struck by foreign objects and affect the robot's movement. Therefore, the Wheel Differential model is selected, and optimized for turning around tight corners and for reducing sliding friction.

**Figure 4:**
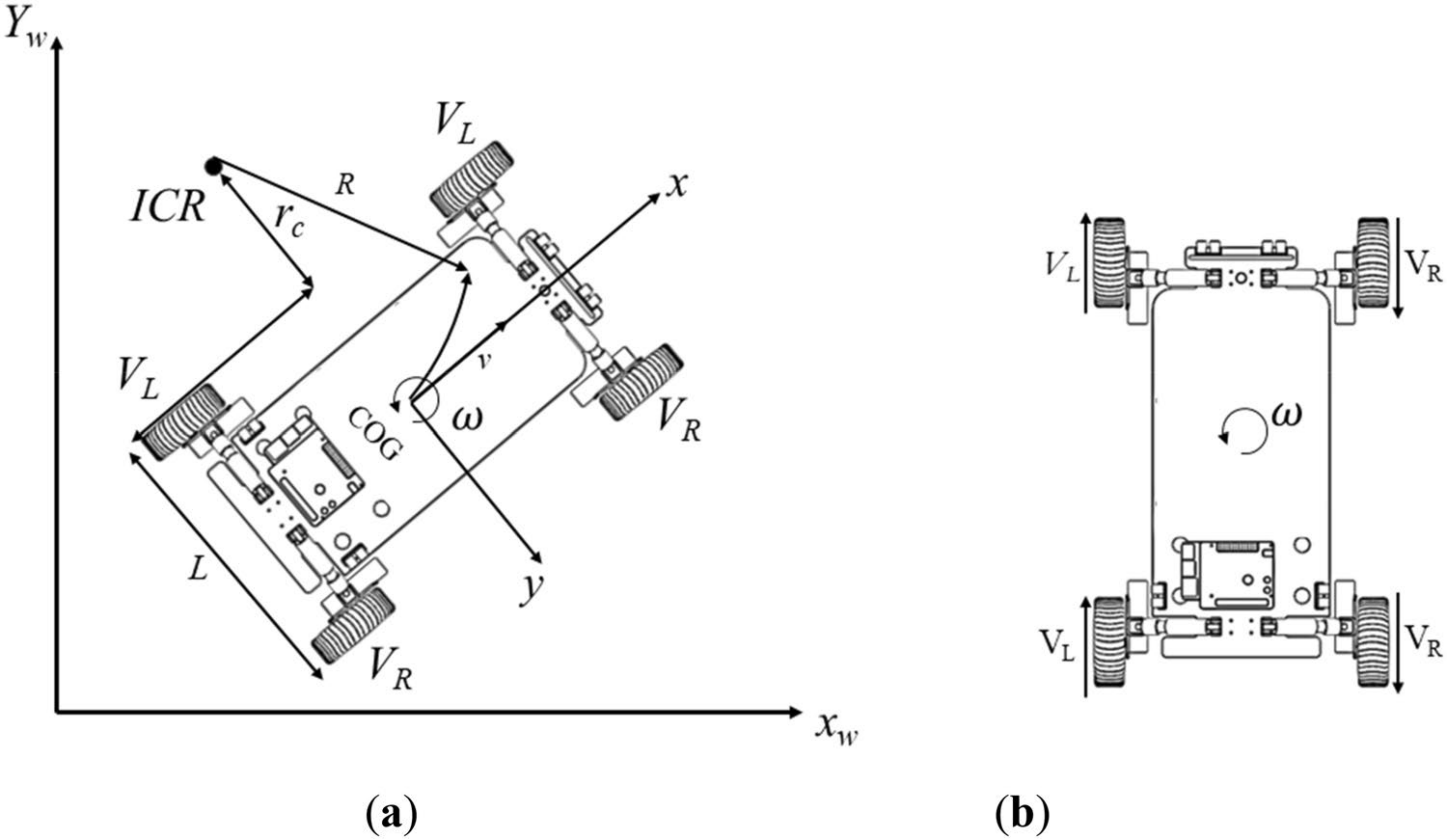
(**a**) Motion model of the differential speed robot. (**b**) The steering model.

For the kinematics of the robot, a local coordinate frame, denoted as (*x*, *y*, *z*), is located at the center of gravity (*COG*) of the model. The motion of the robot is on the horizontal plane, formed by the *Xw* and *Yw* axes of the world coordinate system, shown in Fig. 4a.

The instantaneous center of rotation of the robot is *ICR*, the linear velocity of the left wheel is *V*_*L*_, the linear velocity of the right wheel is *V*_*R*_, the angular velocity is *ω*, the distance between the two wheels is *L*, and the distance from the left wheel to the center of the circle is *r*_*c*_.

The physical relationship between angular velocity, linear velocity (v), and radius of motion of the differential robot is as follows:

1 $$v =\omega \times R$$

The decomposition of the velocity of the left wheel and the right wheel can be found as:

2 $$\left\{\begin{array}{c}{V}_{L}=\omega \times {r}_{c}=v-\frac{L}{2}\omega \\ {V}_{R}=\omega \times {r}_{c}=v+\frac{L}{2}\omega \end{array}\right.$$

From this, the relationship between the overall linear velocity of the robot, the angular velocity, and the left and right wheels can be solved as follows:

3 $$\left\{\begin{array}{c}v=({V}_{L}+{V}_{R})/2\\ \omega =({V}_{R}-{V}_{L})/L\end{array}\right.$$

As shown in Fig. 4b, the left and right wheels of the robot designed are configured in parallel, and the robot turning is realized by the speed difference between the left wheel and the right wheel. The radius of curvature of the turn increases when the speed difference between the left and right wheels is larger. When the robot works in a narrow space, the robot turns around its middle vertical axis, i.e., the left and right wheels have the same speed but in opposite directions, and the turning radius is 0.

**Figure 5:**
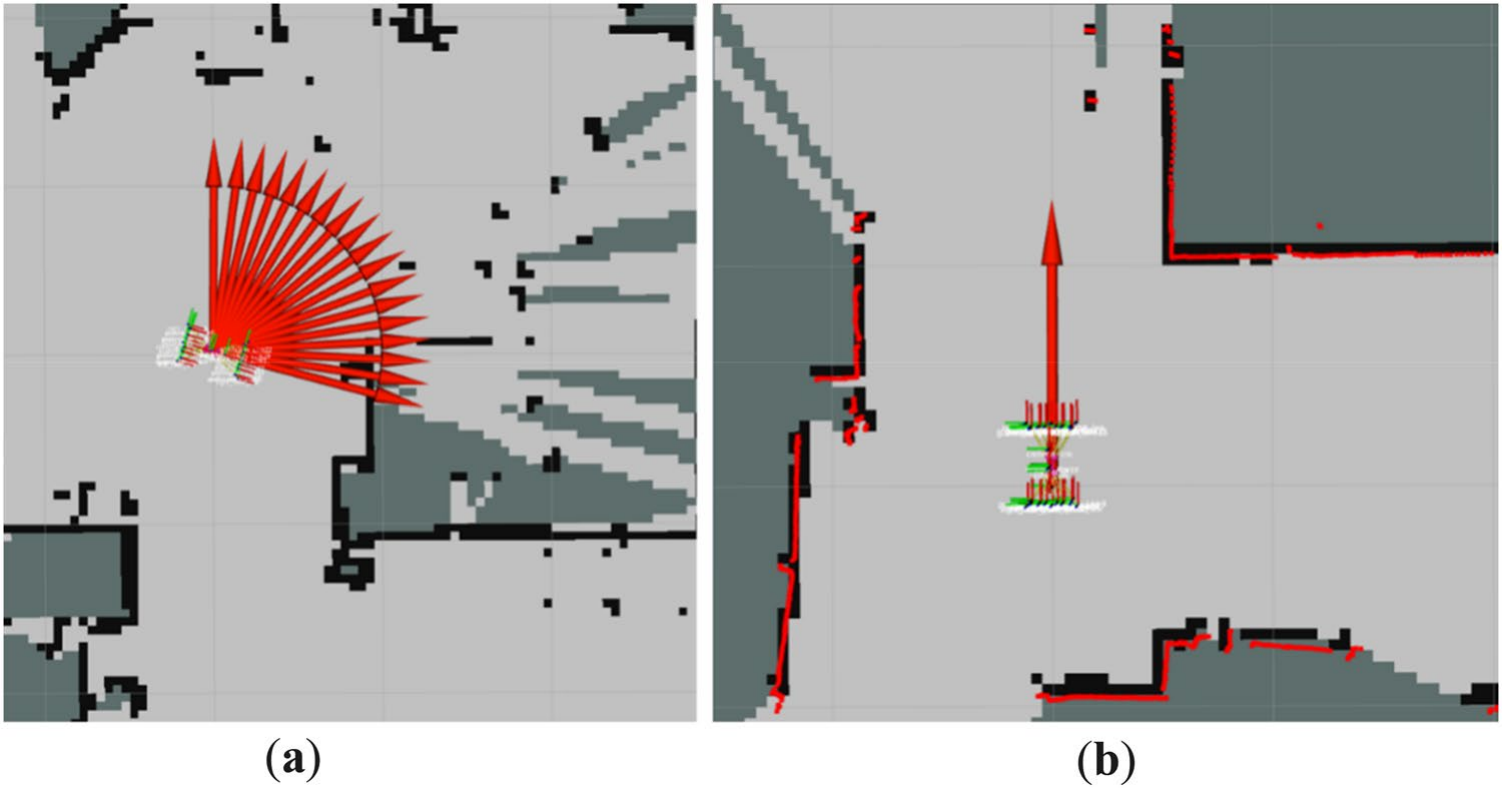
Testing the chassis in a simulation environment: (**a**) Turning test. (**b**) Straight-line speed test.

The performance of key parts of the robot needs to be analyzed using specialized software before it is actually applied in order to avoid unnecessary waste of time and money^17^. In order to investigate the slippage problem of the above motion model, the simulation is carried out in gazebo. The dynamic tag is added to the tire link tag and the ground link tag in the XACRO file of the robot model. A dynamic tag is to add to measure the friction coefficient between the tire and the ground. The parameters are shown in Table 1. Figure 5a shows the rotation speed of the inspection robot around its own central axis. Figure 5b shows the speed test of the inspection robot walking a straight line. Through the simulation, it is found that the motion model can complete going straight and rotating, while the occurrence of slippage is relatively mild.

**Table 1:** Motion model test parameters.

Friction coefficient	Linear velocity along x-axis	Angular velocity around z-axis
0.5	0.3 m/s	1.0 rad/s

### Vision inspection system

**Figure 6:**
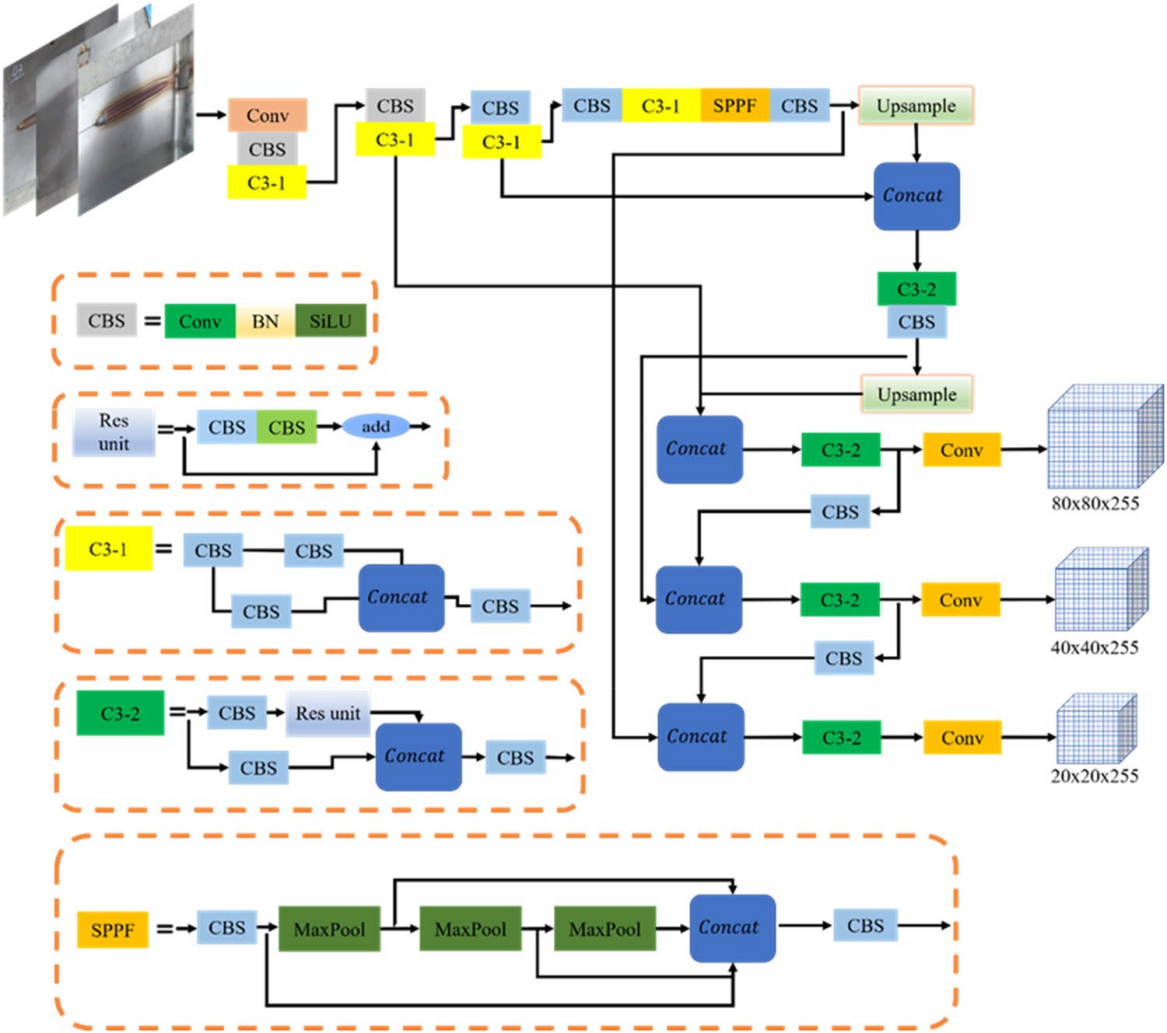
YOLOv5 network structure.

The visual inspection function is for identifying and locating the weld seam. The images are captured at the robot side and the weld seam pictures are identified at the computer side using YOLOv5 inspection algorithm. The YOLOv5 based target detection algorithm, which has the advantages of high detection speed and lightweight deployment model, is used for the robot's inspection system. The YOLOv5 algorithm has four structures (i.e., s, m, l, and x) to represent different depths and widths of the network. It uses indicators to deepen and widen the network, but as the detection accuracy increases, the response velocity becomes progressively slower. The device design in this study requires model lightweight, real-time responses, and any-size image input, so YOLOv5 is chosen as the benchmark model, and its structure is shown in Fig. 6.

#### A brief description of Yolov5s model

The YOLOv5 network structure is consisted of the input, back­bone, neck, and detection end. The input includes the data enhancement of the mosaic, adaptive calculations of the anchor frame and adaptive scaling of the image. The back-bone is mainly composed of the CBS (convolution, BN layer, SiLU activation function), SPPF (spatial pyramid pooling-fast), and C3 (concentrated-comprehensive convolution) modules^18^. Among them, the batch normalization (BN) layer solves the problems of gradient disappearance and gradient explosion through data normalization. The sigmoid weighted linear unit (SiLU) activation function is a smooth and non-monotonic function, and it prevents the gradient from gradually decreasing to 0 during slow training.

The neck is the combination of FPN^19^ and PANet^20^. The deep-feature map contains stronger semantic features and weaker localization information, while the shallow-feature map contains stronger location information and weaker semantic features. FPN transfers the semantic features from the deep-layer to the shallow-layer to enhance the semantic representation at multiple scales, while PANet transfers the location information from the shallow-layer to the deep-layer to enhance localization at multiple scales. PANet adds a bottom-up direction enhancement on top of FPN.

For network training, the loss function plays an important role in the weld detection model, which marks the difference between the predicted and actual values of the model. In YOLOv5s, a joint loss function is used to train bounding box regression, classification, and confidence. The used loss function (*L*_*l*oss_) is as follows^21^:

4 $${L}_{lo{\text{ss}}}={L}_{box}+{L}_{cls}+{L}_{conf}$$

where *L*_*cls*_ indicates the classification error; *L*_*box*_ indicates the bounding box regression error; *L*_*conf*_ indicates the confidence error.

#### Acquisition and annotation of images

The weld seam is inspected and the acquisition photos are of the completed weld seam. The image acquisition device is a camera (China Vistra Q16 2k usb camera) with an f-value of 1.8. Photographs of the weld seam are collected in different environments (partially obscured weld seam, weld seam at various distances from the camera). A total of 300 images of the weld seam are collected at a distance of about 20–30 cm. To speed up the model training, the images are compressed to 512 × 341 pixels, and saved in jpg format. Example images are shown in Table 2. The collected images are annotated using LabeIImg, and the annotation produces xml files for model training. The data are divided into training set, validation set, and test set, with a ratio of 7:2:1, while there are no data duplications among the sets.

**Table 2:** Composition of dataset for training, validation, and test, including assignment of classes and number of images per class.

Set	Weld seam	Number of Images
Training	210	210
Validation	60	60
Test	30	30
Total	300	300

#### Experimental environment and parameter setting

The training model for weld detection is simulated on an HP Shadow Wizard computer with the configurations shown in Table 3.

**Table 3:** Hardware and software versions.

Hardware	Configure	Environment	Version
System	Windows10	Python	3.9.12
CPU	I7-9750H	Pytorch	1.11.0
GPU	GTX 1650 (4G)	Pycharm	2019
RAM	32G	Cuda	11.1
Hard-disk	512G	Cudnn	8.0.4

**Figure 7:**
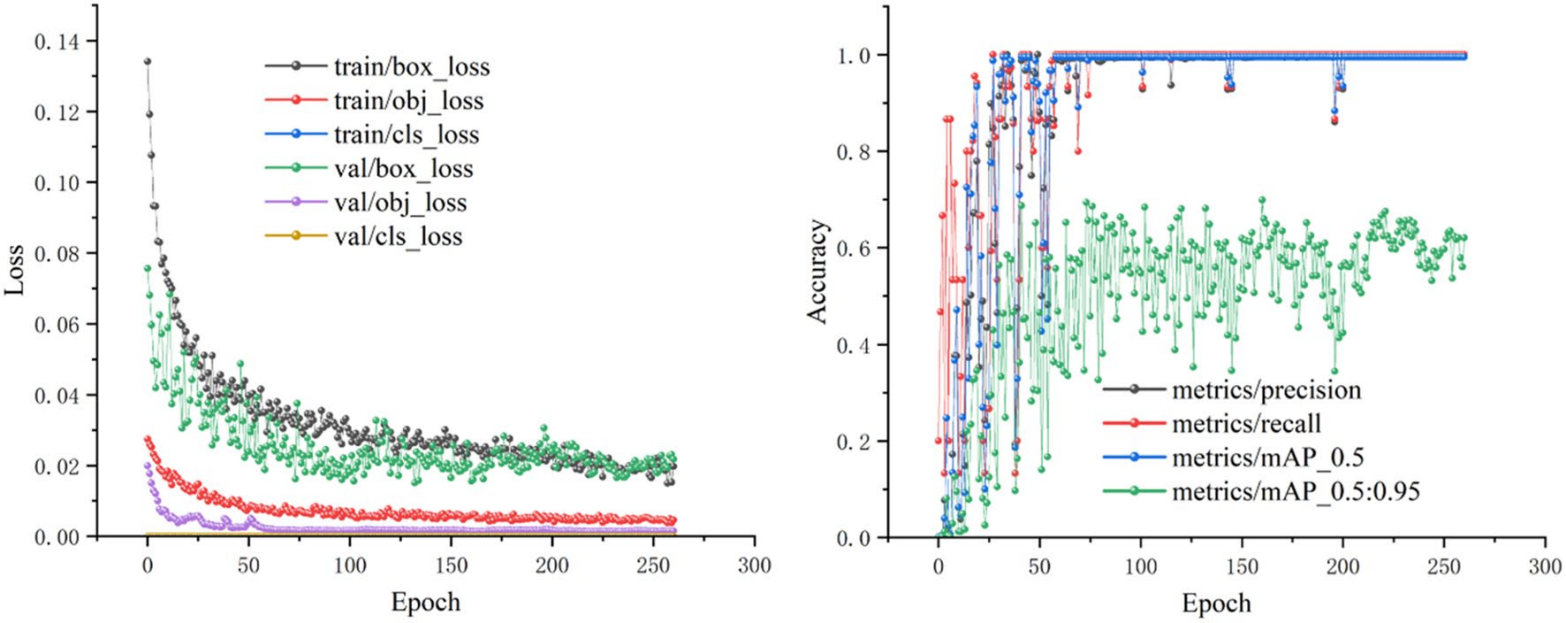
Convergence results of the pre-training model.

The hyperparameters optimized for training using the above hardware are: epoch value is 150, learning rate is 0.01, momentum is 0.937, weight decay is 0.0005, batch size is 16, workers are 8, optimizer is stochastic gradient descent (SGD), and a single graphic processing unit (GPU) is used to speed up the training. All hyperparameters are performed for the pre-training on the Validation set. The change in the loss values of the pre-training process is shown in Fig. 7. It can be seen that the loss value decreases rapidly at the beginning of the training period, and after 50 rounds of training, it tends to be smooth and converges, without any underfitting or overfitting.

## Control architecture

### Electronics and control

**Figure 8:**
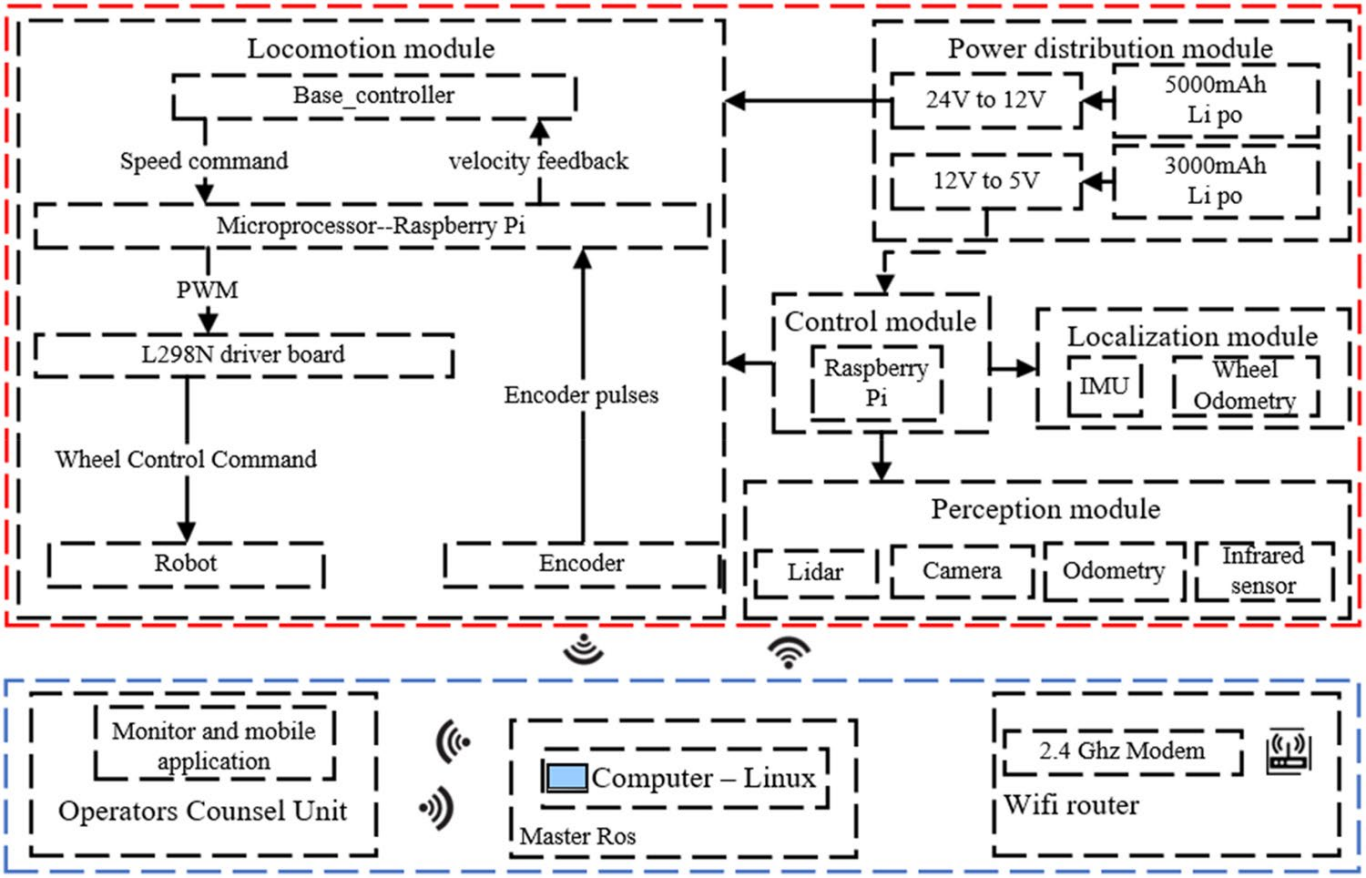
Control system of the robot.

The control architecture of the robot is shown in Fig. 8. The motion module is controlled by four DC motors with an encoder (gear ratio 1:90, size 25 × 25 × 80 mm), two motors are installed on each side of the chassis, and all motors are connected to the L298n motor driver board through a Dupont cable. The motor driver board and the Raspberry Pi main control board are connected through the IO port. The Raspberry Pi subscribes to the data from the encoder through the IO port, and it processes the data and sends speed commands to the motors. The sensing module of the robot consists of the LIDAR (Lidar A1M8), a camera head, an odometer, and an infrared sensor. The laser sensor, communicating with the Raspberry Pi through a USB port, is used for building a map of the surrounding environment, positioning, and avoiding obstacles. However, LIDAR has a blind scanning area, and there is a possibility that obstacles in the complex environment are not perceived, so the infrared sensor is used to supplement and improve the obstacle sensing. The odometer is used for robot positioning, and the LIDAR positioning is used to improve the odometer accuracy. The camera head is connected to the Raspberry Pi via a USB port for weld detection. The power supply module consists of two batteries (12 V 5000 mAh; 12 V 3000 mAh). The 5000 mAh battery supplies power to the motor driver board L298n. The 3000 mAh battery supplies power to the Raspberry Pi mainboard, and the rest of the sensors are powered through a USB or IO port.

The motors do not use stm32 or the Arduino control method, but directly connect to the motor driver board through the IO port on the Raspberry Pi, which improves the convenience of operation and the sensitivity of control. The core controller of the robot is the Raspberry Pi 4B. The data processed on this robot is moderate, so the Raspberry Pi 4B is sufficient, which has Ubuntu 18.04 Linux and Robot Operating System (ROS) installed. The data collected by various sensors are transferred to the ROS system for processing. The various sensors, main control board, GUI, and PC are integrated through the ROS framework. To reduce the pressure on the Raspberry Pi for processing the data, the collected data are transferred through WIFI from Raspberry Pi to the PC, for processing on the distributed framework of ROS.

### Graphical user interface (GUI)

When they are connected to the same network, both the robot and PC can be remotely monitored and controlled through the distributed ROS. Communication between the robot and control device is carried out through ROS' Message Queue Telemetry Transfer (MQTT). When the navigation control node is started on the mobile side, a message is received on the PC, and a map building command can be executed on the PC at the same time, which controls the robot to build a map and issues a point-to-point cruise operation. This operation reduces the computational pressure on the Raspberry Pi 4B onboard the robot. A graphical user interface for monitoring and control is developed for the PC using the qt software. The robot can be viewed through the GUI when it is working in an unknown environment, and is assigned with control buttons and video outputs.

## Navigation and control in complex environments

### Selection of local path planning

Path planning requires the cooperation of global path planning and local path planning. The weight of global path planning in the obstacle avoidance process is less than that of local path planning. Therefore, in this study, the local path planning is emphasized. The most critical issue is that the robot must safely avoid static obstacles and dynamic obstacles during an inspection. The local path planning methods in complex environments include the Artificial Potential Field method (APF)^22^, Genetic Algorithm^23^, Dynamic Window method (DWA) ^24^, Neural Network Algorithm, and other intelligent algorithms. The ATF method tends to fall into local minima and fails to reach the focus position^25^, and the Neural Network algorithm is too demanding on the performance of the main control board^26^. All the above algorithms have a lower convergence speed, and none of them have the ability to avoid local extremes.

An improved TEB algorithm is, therefore, selected to implement the local path planning. The TEB algorithm was proposed by Rösmann ^27^ and was based on the classical elastic band algorithm, which was an obstacle avoidance method by optimizing multi-objective trajectory optimization. Compared with the local path planning algorithms described above, the TEB algorithm can set multiple constraints as needed to ensure the applicability of the algorithm. The multi-objective optimization of the TEB algorithm relies on only a few continuous states, thus optimizing for a sparse matrix model. Rösmann et al. proposed that the sparsity problem of the hypergraph-based TEB algorithm can be solved quickly and efficiently using the G2o framework to improve the computational speed. However, mobile robots equipped with the TEB algorithm can appear to be trapped in local minima and unable to cross obstacles in complex environment navigation. To solve this problem, Rösmann et al. proposed an extension of the TEB technique by using parallel trajectory planning in a spatially unique topology^28,29^. However, these approaches only considered the location of obstacles and did not consider potential collisions between the robot and surrounding obstacles. Lan et al. ^30^ proposed an active timed elastic band (PTEB) technique for autonomous mobile robot navigation systems in dynamic environments. Previous work to improve the effectiveness of TEB algorithms operating in complex environments has focused on obstacle avoidance. However, most of the related research only pursued avoiding local minima and smoothing the planned paths in complex environments. They did not consider the shortest local path, and the planned local path might not be the optimal path^31^. Therefore, the improved TEB algorithm still suffered from the robot backing up during turns, local detours, and unable to enter narrow areas.

The improved TEB algorithm proposed in this study optimizes the behavior of local bypassing and reversing, adds the constraint of angular acceleration to the constraints of the multi-objective optimization, and considers the time consumption brought by excessive turning. The proposed TEB algorithm is proved by experiments to achieve fast turning and reduce the behavior of reversing, improve the detection range of the inspection robot, and reduce the time cost of the inspection.

### Timed elastic band algorithm (TEB) model construction

**Figure 9:**
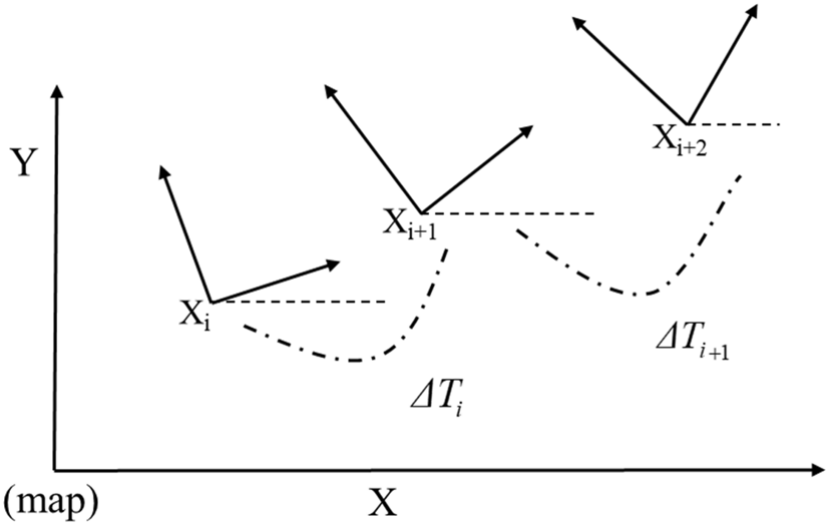
Pose and time interval of mobile robot in the world coordinate system.

The proposed TEB algorithm is based on the elastic-band-algorithm with the addition of temporal information between bit-pose sequences, as shown in Eq. (5), which considers the dynamic constraints of the robot, and modifies the trajectory directly, instead of modifying the path. The operation principle of the TEB algorithm is to convert the position information of the searched initial path into the trajectory sequence with time information for the existing global path points, as shown in Fig. 9. The large-scale optimization algorithm of the sparse system in the "G2O framework" is solved to obtain the optimal control quantity that satisfies the constraints, and the robot drive system is directly commanded by calculating the control variables v and ω, as in Eq. (6):

5 $$\genfrac{}{}{0pt}{}{Q=\left\{{X}_{i}\right\},i={0,1},2\dots n n\in N}{\tau =\left\{{T}_{i}\right\},i={1,2},\dots ,n-1}$$

6 $$B:=(Q,\tau )=[{X}_{l},{\Delta T}_{l},{X}_{2},{\Delta T}_{2},...,{\Delta T}_{n-l},{X}_{n}]$$

where *X*_*i*_ is the poses at the time *I*, and *Q* is the sequence of the poses; *ΔT*_*i*_ is the time interval between adjacent poses, and *τ* is the time interval sequence; the pose sequence and the time interval sequence are combined into a trajectory sequence *B*.

Because the objective function of the TEB algorithm depends on only a few continuous pose states, this leads to a sparse system matrix that represents these constraints as objectives according to a segmented continuous, differentiable cost function. The function penalizes the violation of the constraints that represent the boundaries, as in Eq. (7).

7 $${\mathcal{l}}_{\tau }\left(x,{x}_{r},\varepsilon ,S,n\right)\approx \left\{\begin{array}{c}{\left(\frac{x-\left({x}_{r}-\varepsilon \right)}{S}\right)}^{n}\\ 0\end{array}\right.\genfrac{}{}{0pt}{}{,}{,}\genfrac{}{}{0pt}{}{x>{x}_{r}-\varepsilon }{x\le {x}_{r}-\varepsilon }$$

where *x*_*r*_ is the critical value, *S* is the scaling factor, and *n* is the polynomial coefficient, which usually takes the value of 2; *ε* is a small section of displacement near the critical value.

**Figure 10:**
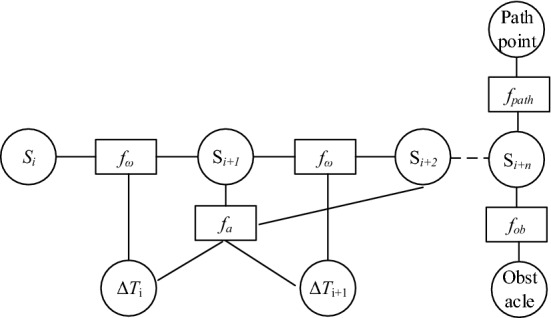
The improved hyper-graph.

The multi-objective optimization function is shown in Eq. (8).

8 $$f\left(B\right)=\sum_{k}{\gamma }_{k}{f}_{k}(B)$$

where *f*_*k*_(*B*) is a constraint function in Fig. 10, and γ_k_ is the weight corresponding to the constraint function.

The trajectory constraints of the TEB algorithm are divided into two parts. The first part is constrained by global path planning; the second part is constrained by velocity, acceleration, and its own kinematic model. In this study, the focus is on optimizing the velocity, acceleration, and obstacle constraints.

The obstacle constraint is the most critical condition to ensure that the robot can avoid the obstacle completely. The minimum distance allowed between the robot and the obstacle is set to *d*_*min*_, and the distance between the robot and the obstacle is set to *D*. The position information of the obstacle on the map is obtained by sensors such as LIDAR. To ensure the safety of the planned trajectory, each bit posed on the TEB trajectory is related to the obstacles appearing on the map, and the penalty function is triggered when the distance *D* between the robot and the obstacle is lower than *d*_*min*_. The penalty function is expressed as Eq. (9):

9 $${f}_{ob}=\sum_{i=0}^{n}\left\{\begin{array}{c}0, {d}_{min}>{d}_{imin}\\ {d}_{min}-{d}_{imin}, {d}_{min}\le {d}_{imin}\end{array}\right.$$

The velocity and acceleration constraints are described similarly for the geometrically constrained penalty functions. The linear and angular velocities are approximated by the Euclidean distance between adjacent poses, and the amount of change in the directional angle can be expressed as Eq. (10).

10 $$\left\{\begin{array}{c}{v}_{i}\approx \frac{1}{\Delta {T}_{i}}\parallel \left(\begin{array}{cc}{x}_{i+1}& -{x}_{i}\\ {y}_{i+1}& -{y}_{i}\end{array}\right)\\ {\omega }_{i}\approx \frac{{\theta }_{i+1}-{\theta }_{i}}{\Delta {T}_{i}}\end{array}\right.$$

The acceleration is related to two consecutive average velocities, so the average velocity corresponding to three consecutive poses needs to be acquired, and can be expressed as Eq. (11).

11 $$\left\{\begin{array}{c}{\alpha }_{i}=\frac{2({v}_{i+1}-{v}_{i})}{\Delta {T}_{i}+\Delta {T}_{i+1}}\\ {\beta }_{i}=\frac{2({\omega }_{i}+1-{\omega }_{i})}{\Delta {T}_{i}+\Delta {T}_{i+1}}\end{array}\right.$$

### Constraints Based on Improved TEB Algorithm

To decrease the energy consumption, the control algorithm for the turning angle speed of the TEB algorithm is considered next. To realize the reverse and detour movtions of the robot in the process of avoiding obstacles, the control algorithm of the angular velocity is optimized. When the target point of the robot is given, the position point of the robot is set to (*x*_*i*_, *y*_*j*_). The adjacent path points are(*x*_*i*_, *y*_*i*_) (*x*_*i*+1_, *y*_*i*+1_), the angle between the line connecting the two points and the robot's initial test pose is *θ*_i_. A minimum threshold angle *θ*_imin_ is set. When *θ*_i_ is greater than the minimum threshold, the angular velocity is set to the maximum. The robot will accelerate its turning to avoid reversing, and as the *θ*_i_ becomes smaller, the angular velocity also decreases to achieve a smooth transition of the turn. The penalty function can be expressed as Eq. (12):

12 $$\upomega =\left\{\begin{array}{c}{\omega }_{max}, {\theta }_{i}\ge {\theta }_{imin}\\ \frac{{\theta }_{i}}{{\theta }_{imin}}\times {\omega }_{max}, {\theta }_{i}<{\theta }_{imin}\end{array}\right.$$

The above angular-velocity optimized control is used as an angular steering constraint, and the angular-velocity constrained edges are added to the hypermesh. A new hypergraph is thus constructed, as shown in Fig. 10. The optimized angular-velocity constraint function is connected to two poses vertices *S*_*i*_ and *S*_*i*+1_. The optimization problem is transformed into a hypergraph, and solved using a large-scale algorithm for sparse systems in the G2O framework. The robot is driven directly by computing control variables *ν *and *ω*.

## Experiments and discussion

### Simulation experiment

The simulation experiments are conducted on the ROS platform, first building the simulation environment in Gazebo, and observing the motion of the robot equipped with the improved TEB algorithm on the RVIZ visualization platform. The motion of the robot is modeled as a 4WD differential, with the left and right wheels controlled separately. The simulation parameters on the simulation platform are given in Table 4.

**Table 4:** Parameter configurations of the simulated experiment.

Constraint Parameters	Values
Maximum × linear velocity (m/s)	0.6
Maximum backward linear velocity (m/s)	0.4
Maximum angular velocity (rad/s)	0.5
Maximum × linear acceleration (m/s2 )	0.5
Maximum angular acceleration (rad/s2 )	0.5
Obstruction expansion radius (m)	0.6

**Figure 11:**
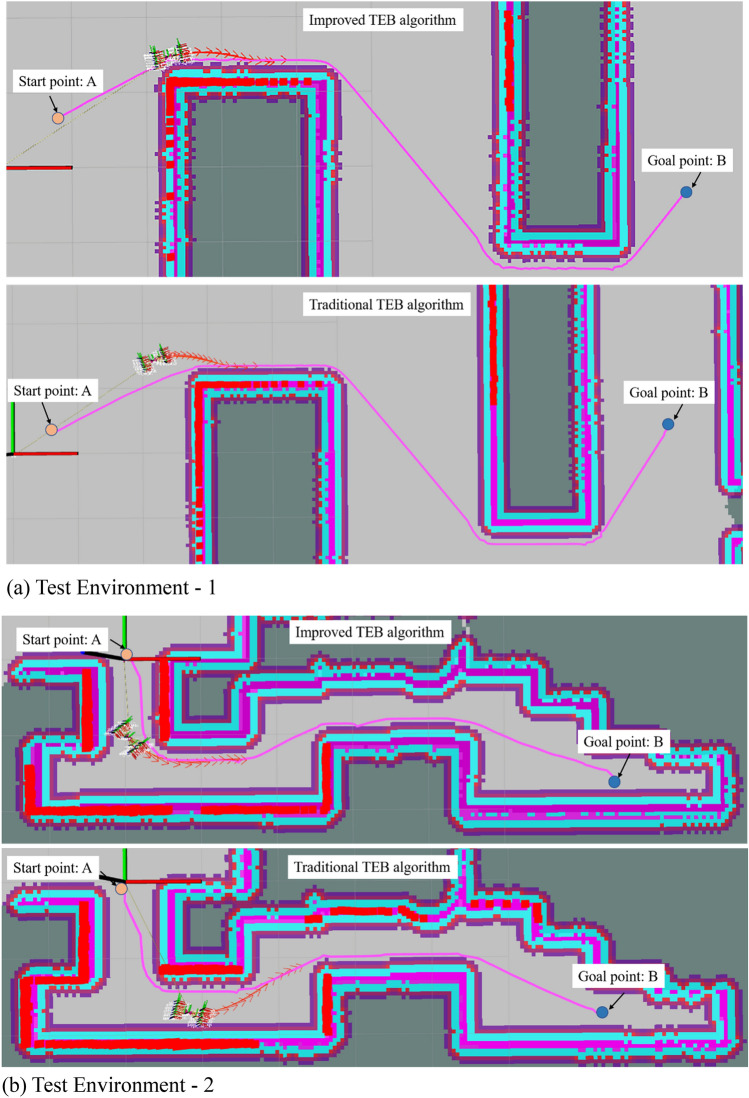
Test environments for robots.

Figure 11 shows the motion process of the improved TEB algorithm and the traditional TEB algorithm in two different environmental scenarios. The robot with the improved TEB algorithm turns in Environment 1 and Environment 2 closely to the global path, with a shortened running time while avoiding the obstacles. While with the traditional TEB algorithm, it is clear that the robot makes greater detours at the turns, increasing the time for a given average speed. As can be seen from Table 5, the running time for the improved algorithm in different scenarios is reduced by about 5 s compared with the running time of the traditional algorithm. The improved TEB algorithm shortens the running time by 12%, ensuring smooth operation and improving efficiency, while the robot moves close to the global path and avoids energy loss due to oversteering.

**Table 5:** Time comparison before and after improving the TEB algorithm.

Test environment	TEB algorithm	Starting time	End time	Total time spent (s)
Test Environment-1	Improved algorithms	10:29:28	10:30:10	42.3
	Traditional Algorithms	10:35:10	10:35:47	37.2
Test Environment-2	Improved algorithms	10:49:08	10:49:29	21.32
	Traditional Algorithms	10:53:22	10:54:22	26.16

**Figure 12:**
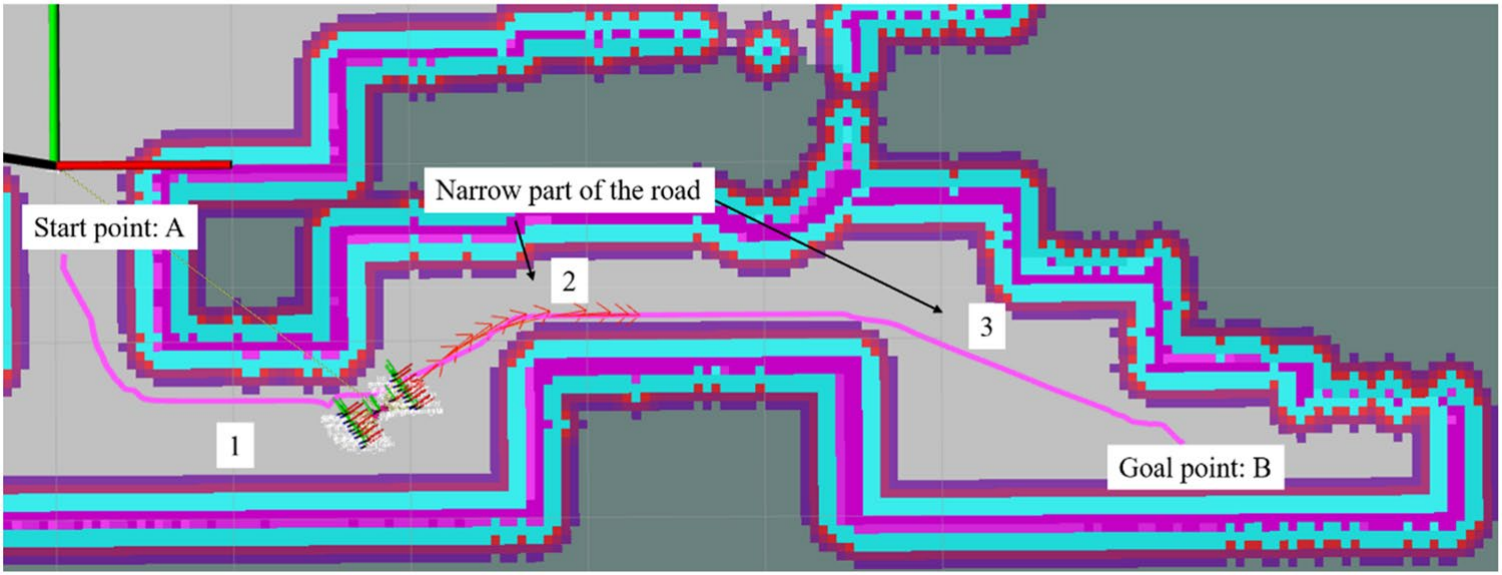
The robot passes through the narrow road.

To verify the turning sensitivity of the inspection robot equipped with TEB algorithm and prevent reversing in narrow spaces, Fig. 12 shows the robot encountering successive narrow road sections. The robot starts from point A through the narrow sections (near the points 1, 2, and 3) to reach point B. The speed profile generated at each stage is viewed to compare the reversing before and after the improvement of the TEB algorithm.

**Figure 13:**
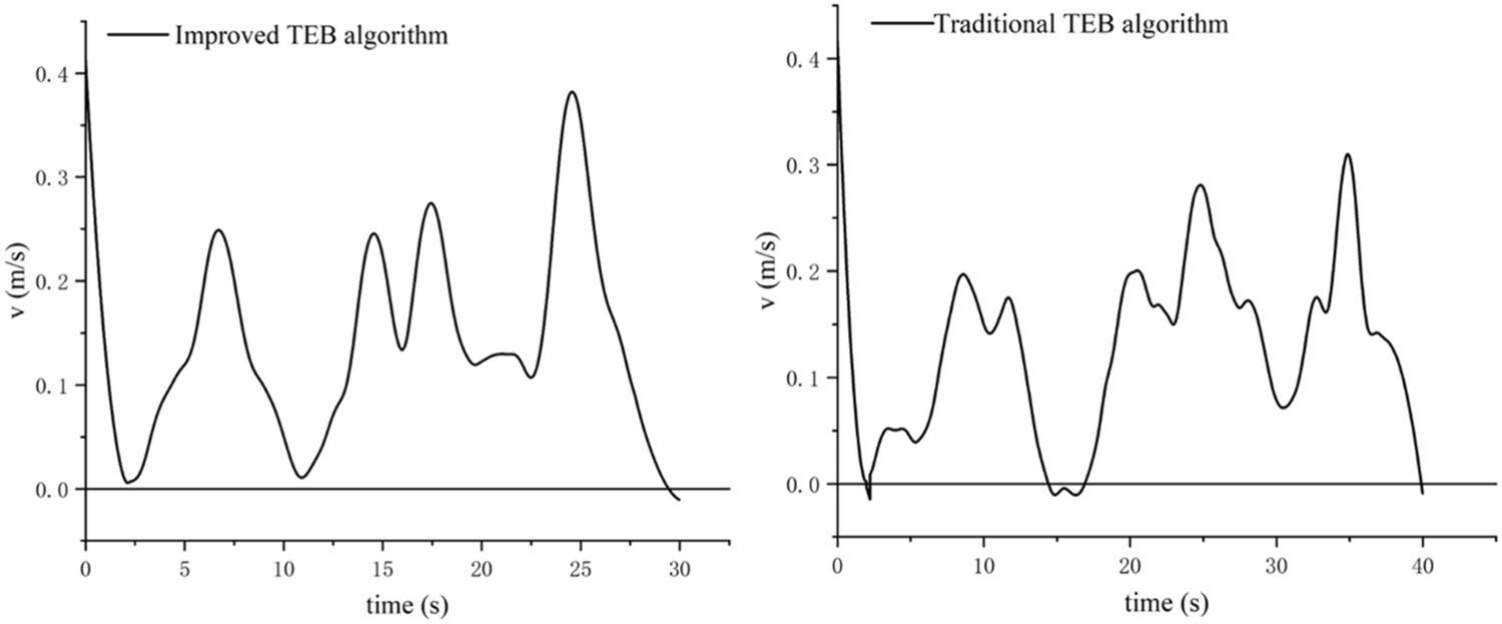
Robot speed output curves of the traditional TEB algorithm and the proposed TEB algorithm.

The velocity output curve of the robot from the starting point A to the target point B, after a continuous narrow road section, is shown in Fig. 13. The traditional TEB algorithm shows reversing phenomena (i.e., the velocity becomes negative) when the robot passes the narrow road sections, where it is very easy to collide; while for the improved TEB algorithm reversing is improved, and turning efficiency, safety, and smoothness are improved. The reversing at the end of both curves is a fine adjustment to get closer to the target point.

### Real robot experiment

The experimental platform of the automatic inspection robot has been built. It is to verify whether the robot's reversal in avoiding obstacles and the robot's turning problem in a narrow environment are significantly improved.

#### Analysis of robot backing behavior in a real-world environment

**Figure 14:**
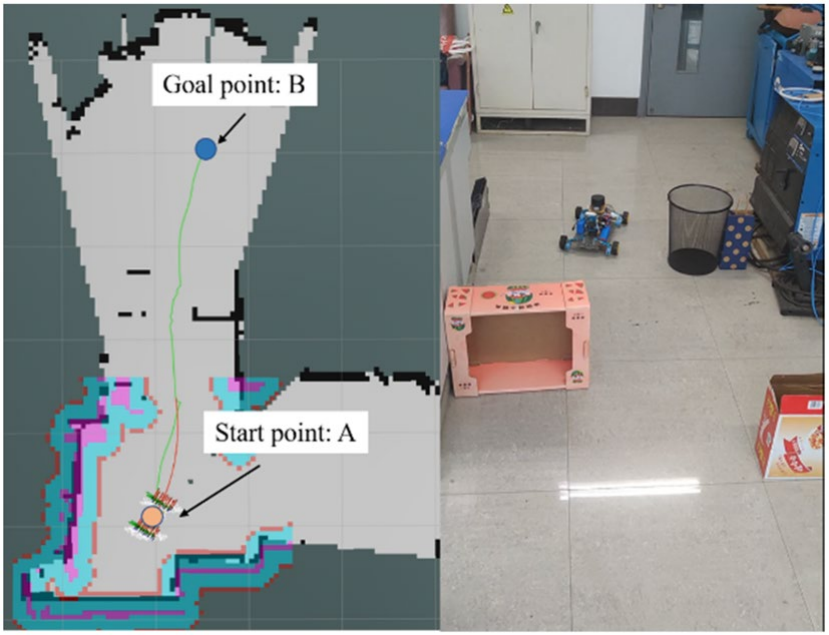
Robot testing environment.

**Figure 15:**
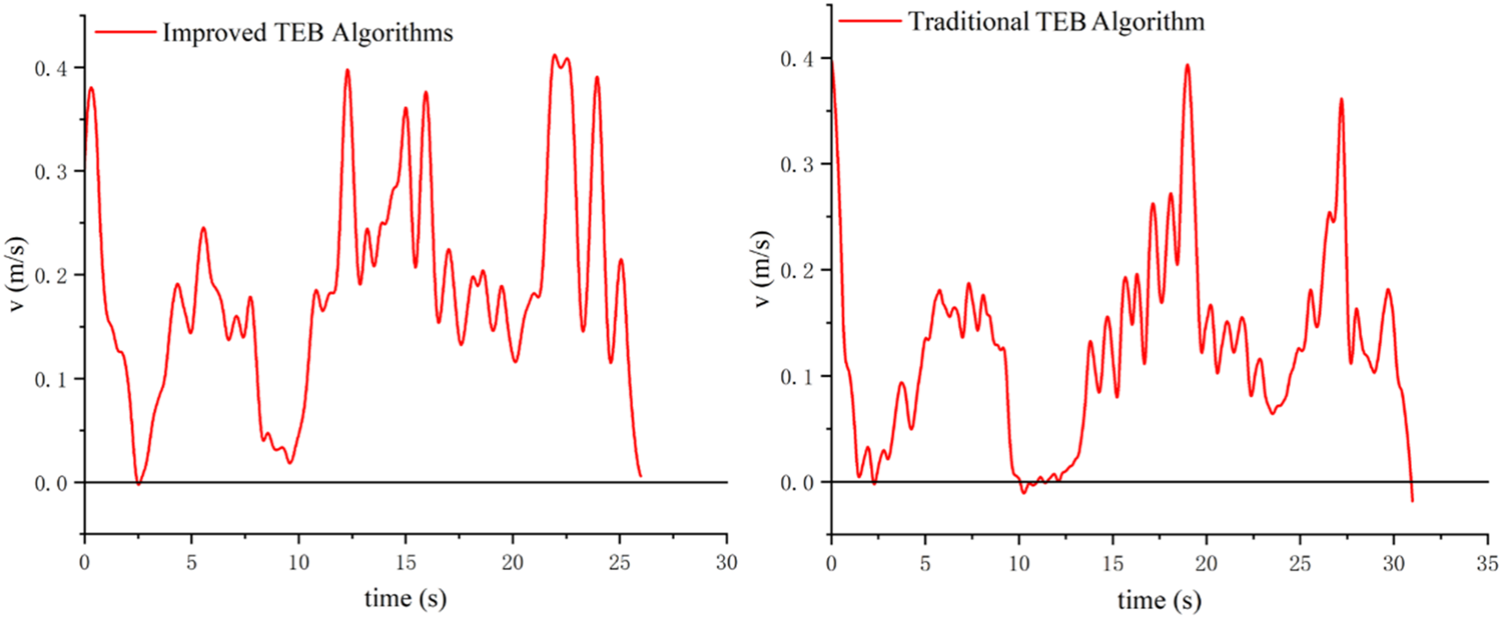
Speed profiles of the robot using the improved TEB algorithm (**a**) and using the traditional TEB algorithm (**b**).

Figure 14 shows the experimental environment for testing the reversing behavior. The actual speed profile of the robot is shown in Fig. 15. It can be seen that the improved algorithm reduces the backing behavior of the robot, and it reduces the running time of the robot. The improved algorithm shortens the running time by about 5 s, and the running efficiency increases by 15%. The actual test results have confirmed earlier simulations.

#### Inspection robot obstacle avoidance motion analysis

**Figure 16:**
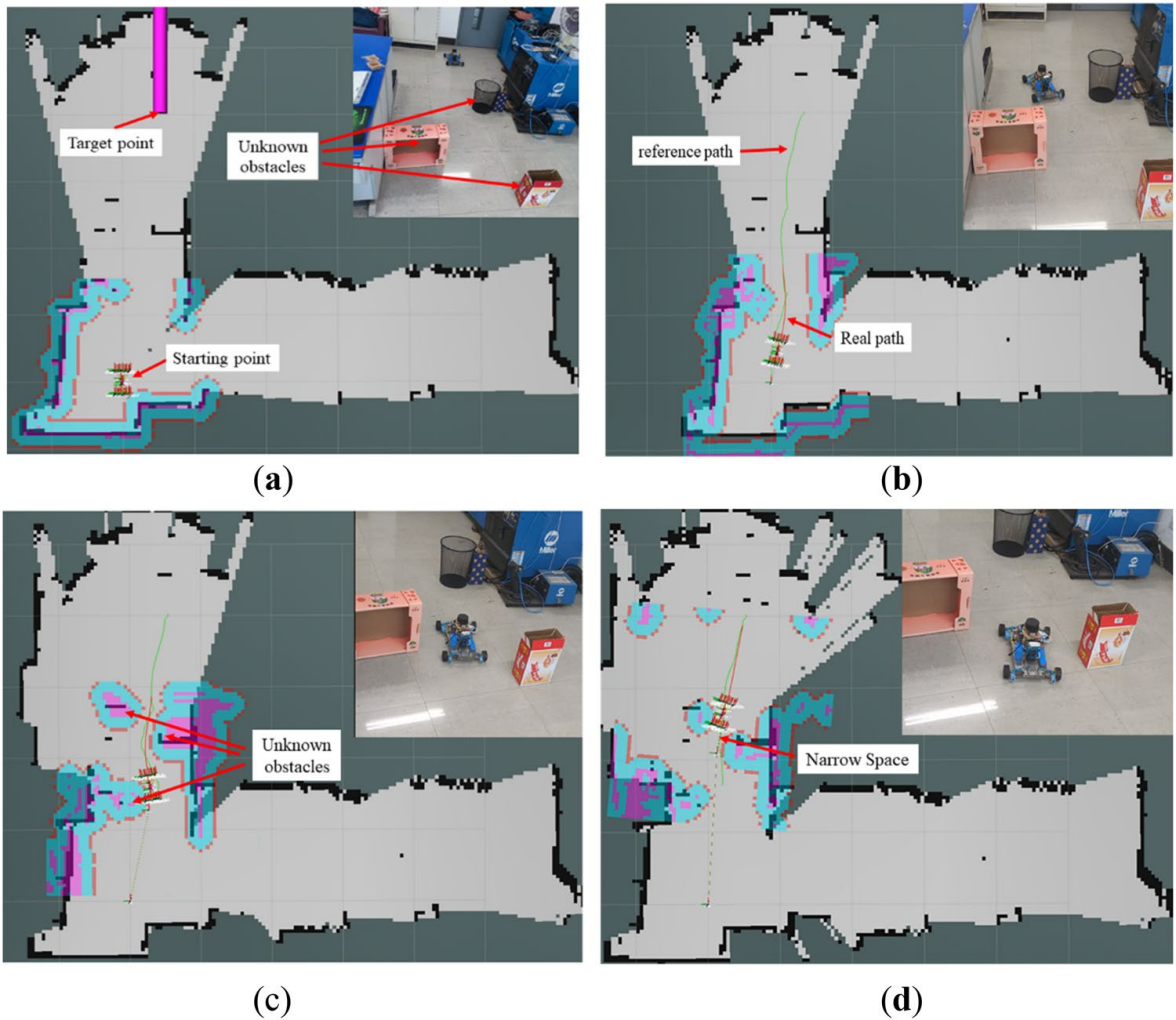
Movement of inspection robots avoiding unknown obstacles and passing through narrow spaces. (**a**) Position of robot and obstacles. (**b**) Robot dodging the first obstacle. (**c**) Detect the second obstacle and re-plan the path. (**d**) Robot passes through narrow space to reach the target position.

Figure 16 shows the actual position of the inspection robot navigating in ROS RVIZ, and with unknown obstacles in a realistic environment. The green line represents the global reference path planned by the robot; the red line represents the real-time planned path of the robot as planned by the TEB algorithm. To test the inspection and the robot's obstacle avoidance in the case of unknown obstacles, the obstacles are not included in the original map. The robot has no prior knowledge of these obstacles, and needs to sense them in real-time during the inspection. There are multiple small boxes that act as static obstacles randomly scattered at different locations to significantly intercept the trajectory of the inspection robot toward the target.

Figure 16a shows the actual positions of the inspection robot and the target. Figure 16b–d show the motion of the inspection robot in encountering unknown static obstacles and successfully reaching the target position in a complex environment. The robot is able to implement the inspection in a complex environment driven by the improved TEB algorithm. In Fig. 16d, the robot passes smoothly in the narrow gap between the obstacles and does not collide with the obstacles and there is no reversed motion. This experiment verifies that the inspection robot can move flexibly in the complex environment, and can plan the path in real-time when encountering obstacles.

#### Weld seam inspection experiment

**Figure 17:**
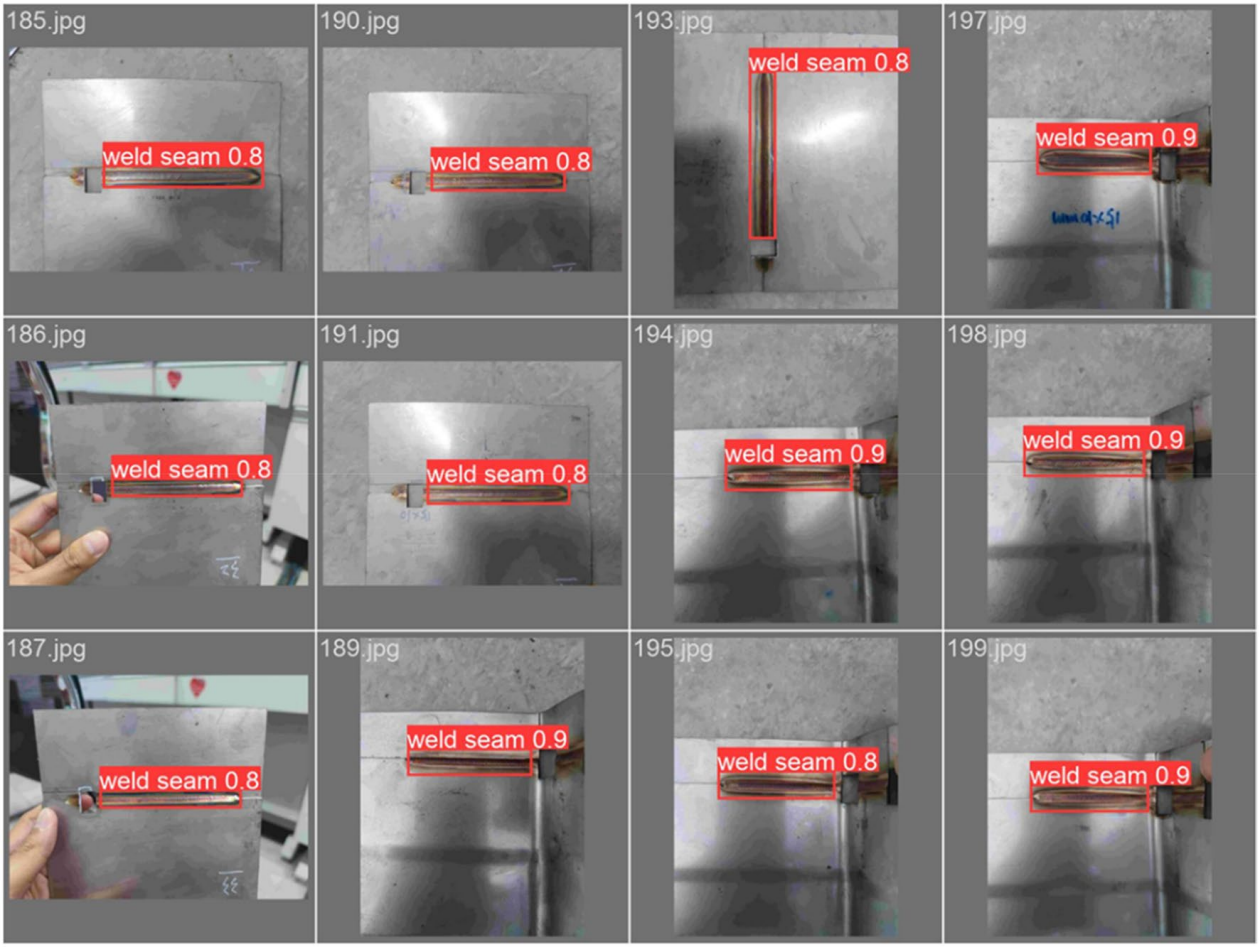
Welding seam test results.

The images collected for this experiment are of the weld seam taken by the robot, and the detection results are shown in Fig. 17. The detection results show that the recognition rate of the detection algorithm is above 90%, which illustrates the effectiveness of the algorithm. The weld seam detection system proposed in this paper is mainly designed to locate and identify the weld seam. After locating the weld seam, the detection system guides the administrator to observe and inspect the weld seam for defects, which is the key part of this study.

This study uses an unmanned vehicle (the robot) with autonomous navigation and obstacle avoidance as a carrier to inspect and locate weld seams using vision detection algorithms. The inspector can set the inspection location as needed, and the robot can independently reach the inspection location, identify and locate the weld seam, and provide inspection information to the inspector. The vast majority of current inspection weld robots^8,9,32^ could only detect weld seams in specific scenarios while requiring staff intervention to adjust the robot's position with real-time monitoring. They relied on the clarity and continuity of the weld seams, and limited span between two weld seams. This study has tested some new ideas and new designs for realistic autonomous weld seam inspections.

## Conclusion

This paper presents the design of a novel flexible inspection robot. The inspection robot is equipped with a four-wheel independent suspension adapted to undulating sections of the ground, a flexible inspection head that can detect all around, and a control algorithm that can detect in narrow passages, all of which improve the efficiency of the robot's inspection. Detection route planning is simulated by an improved Timed-Elastic-Band (TEB) algorithm. Experiments on the path planning algorithm, a key problem for the robot, show that the improved planning algorithm can effectively control the robot in a narrow space, ensuring that the robot does not encounter other obstacles and that the running time is shortened by 12%; the target detection algorithm of yolov5s is also used to train the weld seam detection model with a detection accuracy of better than 90%, based on the robot-provided photo information to identify and locate the weld seam, and provide information to the weld inspector. There is a shortcoming in this study, that the robot recognizes only a single type of weld, and does not detect and classify weld defects.

## Data Availability

The data used in the manuscript are available from the corresponding author on reasonable request.

## References

[1] Salama S, Hajjaj H, Khalid IB (2018). Design and development of an inspection robot for oil and gas applications. Int. J. Eng. Technol. (IJET).

[2] Feng X (2020). Application of wall climbing welding robot in automatic welding of island spherical tank. J. Coastal. Res..

[3] Nguyen L, Miro JV (2020). Efficient evaluation of remaining wall thickness in corroded water pipes using pulsed eddy current data. IEEE Sens..

[4] Hillenbrand C, Schmidt D, Berns K (2008). CROMSCI: Development of a climbing robot with negative pressure adhesion for inspections. Ind. Robot..

[5] Shang J, Bridge B, Sattar T, Mondal S, Brenner A (2008). Development of a climbing robot for inspection of long weld lines. Ind Robot..

[6] Fischer W (2010). Foldable magnetic wheeled climbing robot for the inspection of gas turbines and similar environments with very narrow access holes. Ind. Robot..

[7] Okamoto J (2012). Development of an autonomous robot for gas storage spheres inspection. J. Intell. Robot. Syst..

[8] Wang Y (2019). Design and adsorption force optimization analysis of TOFD-based weld inspection robot. J. Phys. Conf. Ser..

[9] Li J, Li B, Dong L, Wang X, Tian M (2022). Weld seam identification and tracking of inspection robot based on deep learning network. Drones.

[10] Nitta Y, Nishitani A, Iwasaki A, Watakabe M, Inai S, Iwao O (2013). Damage assessment methodology for nonstructural components with inspection robot. Key Eng. Mater..

[11] Krenich S, Urbanczyk M (2012). Six-legged walking robot for inspection tasks. Solid State Phenom..

[12] Bruzzone L, Fanghella P (2016). Functional redesign of Mantis 2.0, a hybrid leg-wheel robot for surveillance and inspection. J. Intell. Robot Syst..

[13] Kim SH, Choi HH, Yu YS (2016). Improvements in adhesion force and smart embedded programming of wall inspection robot. J. Supercomput..

[14] Sun J, Li C, Wu XJ, Palade V, Fang W (2019). An effective method of weld defect detection and classification based on machine vision. IEEE Trans. Ind. Inform..

[15] Li Y, Hu M, Wang T (2020). Weld image recognition algorithm based on deep learning. Int. J. Pattern Recognit..

[16] Yang L, Wang H, Huo B, Li F, Liu Y (2021). An automatic welding defect location algorithm based on deep learning. NDT E Int..

[17] Shanmugasundar G, Sivaramakrishnan R, Venugopal S (2013). Modeling, design and static analysis of seven degree of freedom articulated inspection robot. Adv. Mat. Res..

[18] Li S, Zhang S, Xue J, Sun H (2022). Lightweight target detection for the field flat jujube based on improved YOLOv5. Comput. Electron. Agricult..

[19] Lin, T., Dollár, P., Girshick, R., He, K., Hariharan, B., & Belongie, S. Feature pyramid networks for object detection. in *Proceedings of the IEEE Conference on Computer Vision and Pattern Recognition.* 2117–2125. (2017)

[20] Liu, S., Qi, L., Qin, H., Shi, J., & Jia, J. Path aggregation network for instance segmentation. in *Proceedings of the IEEE Conference on Computer Vision and Pattern Recognition*. 8759–8768 (2018)

[21] Li J, Qiao Y, Liu S, Zhang J, Yang Z, Wang M (2022). An improved YOLOv5-based vegetable disease detection method. Comput. Electron. Agric..

[22] Chen W, Wu X, Lu Y (2015). An improved path planning method based on artificial potential field for a mobile robot. CIT.

[23] Ding S, Su C, Yu J (2011). An optimizing BP neural network algorithm based on genetic algorithm. Artif. Intell. Rev..

[24] Bounini, F., Gingras, D., Pollart, H. & Gruyer, D. Modified artificial potential field method for online path planning applications. in *IEEE Intelligent Vehicles Symposium**Proceedings*. 180–185. 10.1109/IVS.2017.7995717 (2017)

[25] Seddaoui A, Saaj CM (2021). Collision-free optimal trajectory generation for a space robot using genetic algorithm. Acta Astronaut..

[26] Saranrittichai, P., Niparnan, N. & Sudsang, A. Robust local obstacle avoidance for mobile robot based on dynamic window approach. in *2013 10th International Conference on Electrical Engineering/Electronics, Computer, Telecommunications and Information Technology,**Krabi, Thailand.*1–4 10.1109/ECTICon.2013.6559615 (2013)

[27] Rösmann, C., Feiten, W., Wösch, T., Hoffmann, F. & Bertram, T. Trajectory modification considering dynamic constraints of autonomous robots. in* ROBOTIK 2012; 7th German Conference on Robotics,**Munich, Germany*. 1–6 (2012).

[28] Rösmann C, Hoffmann F, Bertram T (2017). Integrated online trajectory planning and optimization in distinctive topologies. Robot. Auton. Syst..

[29] Rösmann, C., Oeljeklaus, M., Hoffmann, F. & Bertram, T. Online trajectory prediction and planning for social robot navigation. in *2017 IEEE International Conference on Advanced Intelligent Mechatronics (AIM)*, *Munich, Germany.* 1255–1260. 10.1109/AIM.2017.8014190 (2017).

[30] Nguyen, L. A., Pham, T. D., Ngo, T. D. & Truong, X. T. A proactive trajectory planning algorithm for autonomous mobile robots in dynamic social environments. in *2020 17th International Conference on Ubiquitous Robots (UR)**Kyoto, Japan*. 309–314. 10.1109/UR49135.2020.9144925 (2020).

[31] Wu J, Ma X, Peng T, Wang H (2021). An improved timed elastic band (TEB) algorithm of autonomous ground vehicle (AGV) in complex environment. Sensors..

[32] Giang, H. N., Anh, N. K., Quang, N. K. & Nguyen, L. An inspection robot for detecting and tracking welding seam. in *2021 Innovations in Intelligent Systems and Applications Conference (ASYU)*. 1–6. 10.1109/ASYU52992.2021.9599065 (2021)

